# Pure & crystallized 2D Boron Nitride sheets synthesized via a novel process coupling both PDCs and SPS methods

**DOI:** 10.1038/srep20388

**Published:** 2016-02-04

**Authors:** Sheng Yuan, Sébastien Linas, Catherine Journet, Philippe Steyer, Vincent Garnier, Guillaume Bonnefont, Arnaud Brioude, Bérangère Toury

**Affiliations:** 1 Laboratoire des Multimatériaux et Interfaces, CNRS, UMR 5615, Université Lyon 1, Université de Lyon, F-69622, Villeurbanne, France; 2 Matériaux Ingénierie et Science, UMR CNRS 5510, INSA de Lyon, Université de Lyon, F- 69621, Villeurbanne, France

## Abstract

Within the context of emergent researches linked to graphene, it is well known that h-BN nanosheets (BNNSs), also referred as 2D BN, are considered as the best candidate for replacing SiO_2_ as dielectric support or capping layers for graphene. As a consequence, the development of a novel alternative source for highly crystallized h-BN crystals, suitable for a further exfoliation, is a prime scientific issue. This paper proposes a promising approach to synthesize pure and well-crystallized h-BN flakes, which can be easily exfoliated into BNNSs. This new accessible production process represents a relevant alternative source of supply in response to the increasing need of high quality BNNSs. The synthesis strategy to prepare pure h-BN is based on a unique combination of the Polymer Derived Ceramics (PDCs) route with the Spark Plasma Sintering (SPS) process. Through a multi-scale chemical and structural investigation, it is clearly shown that obtained flakes are large (up to 30 μm), defect-free and well crystallized, which are key-characteristics for a subsequent exfoliation into relevant BNNSs.

Thanks to their remarkable potential for electronic applications, 2D-nanomaterials related researches are currently booming. Especially, hexagonal boron nitride (h-BN) is a key material in particular within the context of emergent researches linked to graphene. Indeed, it is well known that BNNSs have been considered as one of the best candidates for replacing SiO_2_ as dielectric support or capping layers for graphene^1^. The most important reason is linked to SiO_2_ surface imperfections (roughness, charged traps …), which can significantly limit the carriers’ mobility within graphene’s atomic sheets, then affecting their electronic transport properties. Since lattice parameters of BNNSs perfectly match the ones of graphene, this drawback should be overcome^2^. Recent works^3^,^4^ even present the possibility of building multi-layered hetero-structures in which h-BN and exfoliated graphene flakes are alternatively stacked to form vertically-oriented graphene-based transistors^5^,^6^.

To succeed in these domains of applications, the development of an accessible resource of pure and highly crystallized h-BN crystals remains challenging. Today, only two main sources of hexagonal boron nitride (h-BN) crystals are commonly available for the production of BNNSs after a mechanical or chemical cleavage. Firstly, commercial sources^7^,^8^, generally obtained from an oxygen-containing boron compound reacting with a nitrogen-containing source, which are most often characterized by a high level of defects with relatively small crystalline areas (from less than 2 μm up to 10 μm). Secondly, h-BN-crystals can also be found using the High Pressure High Temperature (HPHT) method adopted at the National Institute for Materials Science (NIMS Japan)^9^,^10^. This process allows the cleavage of relatively large (~100 μm) and thin (several nm) BN samples with an atomically flat surface and low defects density. However, widespread of this latter resource is hindered by its severe production conditions, *e.g.* high temperature (up to 2100 °C), high pressure (5.5 GPa) and treatments time (80 h). Those specificities do not allow, up to now (to our knowledge), the reproducibility of that route by other group.

In order to reach large and highly crystallized h-BN sheets, two strategies can be considered. The first one consists in a direct deposition of the required BNNSs on a substrate, while the second one is based on the generation of BNNSs by exfoliation of large h-BN single crystals.

Considering the first approach, literature reports CVD and CVD-derivatives^11^,^12^,^13^,^14^,^15^ syntheses of BN nanolayers deposited on different substrates using boron and nitrogen based precursors^16^,^17^,^18^,^19^. However, adopting such gaseous-based methods, it is difficult to control the defects formation and to tune the number of layers. The second approach takes advantage of the weak h-BN inter-planar force to get, by exfoliation, a two-dimensional few layers graphene-like structure (often referred as BN nanosheets). Exfoliation is well-documented in the literature and can be performed by mechanical^16^,^20^,^21^,^22^,^23^,^24^ or chemical^25^,^26^,^27^,^28^,^29^,^30^,^31^,^32^,^33^,^34^,^35^ methods. As a consequence, this indirect way for generating BNNSs by exfoliation from high quality h-BN crystals seems more relevant, but actually suffers from a lack of pure h-BN sources of supply, as discussed above.

Keeping this issue in mind, we propose an innovative alternative experimental procedure, which can be widely applied to produce important amount of h-BN flakes with large-scale (>10 μm) and high purity based on an original combination of two advanced techniques: the Polymer Derived Ceramics (PDCs) route, and the Spark Plasma Sintering (SPS) process.

In a first step, the PDCs route is involved in the preparation of very pure preceramic polymer from a molecular resource. Among all available precursors for producing h-BN from the PDCs route, recent works have shown that polyborazylene (PBN) was the best candidate^36^,^37^,^38^,^39^,^40^, due to its easy and reproducible synthesis, combined with its relatively high ceramic yield and high purity. Moreover, the B/N ratio within the polymer is ideal to get further stoichiometric h-BN and there is no contaminant except hydrogen atoms that are easily removed during the ceramization step. In other words, the hexagonal arrangement of h-BN is already present within the molecular precursor’s structure in the borazinic ring. Further developments have shown that an incorporation of lithium nitride as crystallization promoter into the polymeric precursor noticeably improves, from outstandingly low temperatures (1000 °C), both the crystallization rate and the crystallinity level^41^. Recently, we have also demonstrated that, without any sintering step, a simple and classical resistive thermal treatment (<1200 °C) of this additivated preceramic polymer led to a highly crystallized h-BN, prone to be easily exfoliated to h-BN graphene like few layers^42^. In this previous study, the obtained BNNSs displayed size of couple of micrometers, which is not enough to envisage the above-mentioned applications.

The preceramisation process gives rise to an amorphous BN powder. With the purpose to favour crystallisation and increase crystals size, powder is then sintered by SPS^43^. Traditionally, SPS is used to get very dense material. Compared to conventional sintering techniques, near-theoretical density is indeed achieved at lower sintering temperature and for shorter treatment durations^44^. Even if the SPS process is most often applied for a densification purpose, it is known that its parameters^45^ (current pulse, pressure, heating rate) may also influence nucleation-growth mechanisms^46^. It is reasonable to assume that the intense pulse current lines generated during the SPS process may modify the crystallization step promoting the crystals growth. In that direction, the synergy between PDCs and SPS is a promising and accessible strategy to get pure, large h-BN flakes. The targeted BNNSs could then result in a subsequent exfoliation step.

## Results and Discussion

### Bulk sample

**Figure 1:**
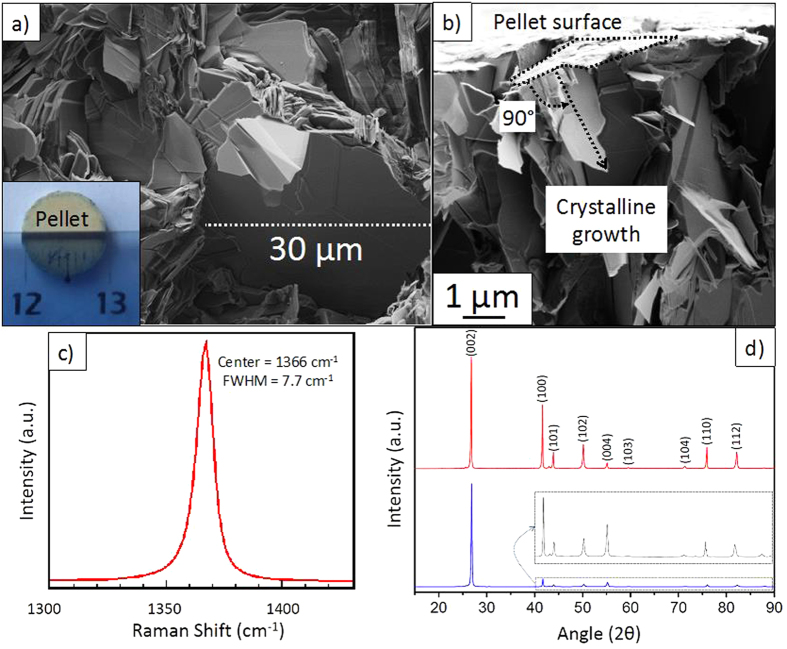
(**a**) SEM image showing stacked layers with micrometric sizes, inset: 1 cm-diameter pellet; (**b**) SEM image showing the pellet cross section and BNNSs aligned perpendicularly to the surface pellet; (**c**) Raman scattering spectrum recorded on the raw sample and (**d**) XRD patterns obtained with the raw sample (upper) analysis of the pellet surface and (bottom) analysis of the pellet cross-section.

**Figure 2:**
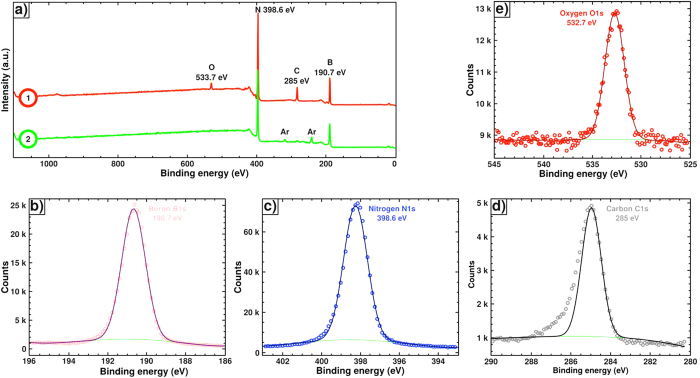
(**a**) XPS general surveys obtained on the raw sample (1) and after 1-μm abrasion (2); (**b**) B1s peak and the corresponding deconvolution recorded on the raw sample; (**c**) N1s peak and the corresponding deconvolution recorded on the raw sample; (**d**) C1s peak and the corresponding deconvolution recorded on the raw sample and (**e**) O1s peak and the corresponding deconvolution recorded on the raw sample.

Liquid PBN is first mixed with Li_3_N micro powder (5 wt. %) and preceramized at 650 °C prior to be sintered in the SPS apparatus. After 1 h under optimized experimental conditions (90 MPa pressure, 1800 °C, constant current of 770 A, 10 ms pulses ON and 5 ms OFF, vacuum in the SPS chamber of 0.15 mbar) a white pellet of 1 cm in diameter is obtained (Fig. 1a inset). A preliminary characterization of the chemical bonding within the specimen is investigated by X-ray photoelectron spectroscopy (XPS). Two analyses are performed on the same sample before and after an Ar^+^ sputtering of the surface, leading to 1 μm depth abrasion. Both general surveys appear on Fig. 2. Photoelectron peaks from B1s, N1s, O1s and C1s are clearly recognized in the spectrum recorded on the raw sample, giving an elemental chemical composition of 42.5; 43.6; 2.4 and 11.5%at., respectively. The binding energies of the B1s and N1s, at 190.7 eV and 398.6 eV, respectively, are consistent with reported XPS data for BN^47^. Both peaks are perfectly symmetric, confirming that B and N atoms are exclusively involved in BN bonds^48^,^49^. Moreover, the carbon peak at 285.0 eV, attributed to a pollution source, is very common with XPS measurements. The asymmetric shape of the C1s peak, typical of carbon pollution, still emphasizes this interpretation^50^. In the same way presence of the slight amount of oxygen (signal at 532.7 eV) would be linked to an oxygen impurity probably resulting from residual water traces or from oxygen adsorbed at the sample surface. However, this first analysis gives an elemental B/N ratio of 0,97, i.e. very close to the one of stoichiometric BN. The second analysis performed after abrasion (4 keV Ar^+^ sputtering), allows confirming that carbon and oxygen resulted from extrinsic pollution since they are almost absent in the sample (<0.5%at. for O1s and 1.3%at. for C1s). After etching, determination of the B/N ratio is more complicated since a preferential sputtering of nitrogen atoms occurs, leaving behind a boron-rich surface (54.6%at. for B1s vs 44.1%at. for N1s)^51^.

The as-sintered sample is then characterized adopting a multiscale approach (Scanning Electron Microscopy (SEM), Raman spectroscopy and X-Ray Diffraction). Figure 1a presents a representative SEM image recorded on the raw sample. It clearly shows very large and homogenous BN domains (up to 30 μm) well staked into differentiated flakes. If the pristine bulk pellet is clearly polycrystalline, we will show that each flake is a single crystal. Furthermore, it can be highlighted that BN flakes have a preferential orientation perpendicularly to the pellet surface e.g. aligned in the compressed direction (Fig. 1b). Figure 1c presents the Raman spectrum recorded on the raw sample. It is well known that Raman peak position and Full Width at Half Maximum (FWHM) are both parameters relative to crystallinity level of the sample. We can observe a well-defined single peak at 1366 ± 1 cm^−1^, which is characteristic of the E_2g_ vibration mode of h-BN crystal^40^. This value is very close to the theoretical one reported for h-BN bulk material at 1367 cm^−1^ 52. The slight difference with the bulk reference material is due to residual inter and/or intragranular stresses developed into the pellet and may result from the high energetic sintering process. This peak is symmetric with a FWHM of 7.7 cm^−1^. To the best of our knowledge, such a value is comparable to the one reported for the best h-BN single crystals obtained by HPHT^9^,^10^,^53^,^54^. This result is correlated with a very low defect density and a very large crystallite size. Figure 1d presents two XRD patterns performed on the raw sample. The upper spectrum is an X-Ray analysis of the pellet surface, and the other one is an X-ray analysis of the pellet cross-section. Both patterns exhibit thin, intense and well-separated peaks indicating a good structural ordering. All peaks have been assigned to expected crystallographic planes from an h-BN crystal according to the JCPDS file number 01-073-2095 (Table 1). The comparison of both patterns clearly shows a drastic decrease of the intensity of all peaks except the {001} ones pointing out a preferential orientation of the h-BN crystallites along the *a* axis (characteristic of the covalent B-N bond) parallel to the applied load.

In summary, large micrometers-crystallized domains (up to 30 μm) grew in the sintering load direction, thus validating our method strategy based on the coupling of PDCs and SPS methods.

## Boron Nitride NanoSheets (BNNSs). 

### Mechanical exfoliation

**Figure 3:**
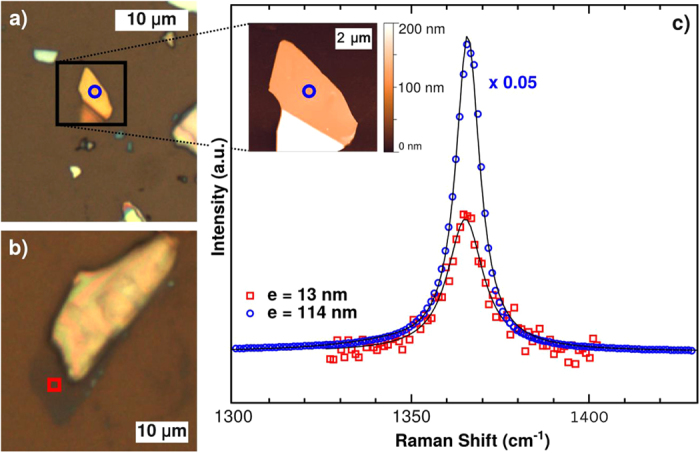
(**a,b**) optical images of BNNSs with different thicknesses deposited on Si0_2_ surface and (**c**) Raman spectra recorded on the corresponding BNNSs, inset: AFM image obtained on the thicker sample.

**Figure 4:**
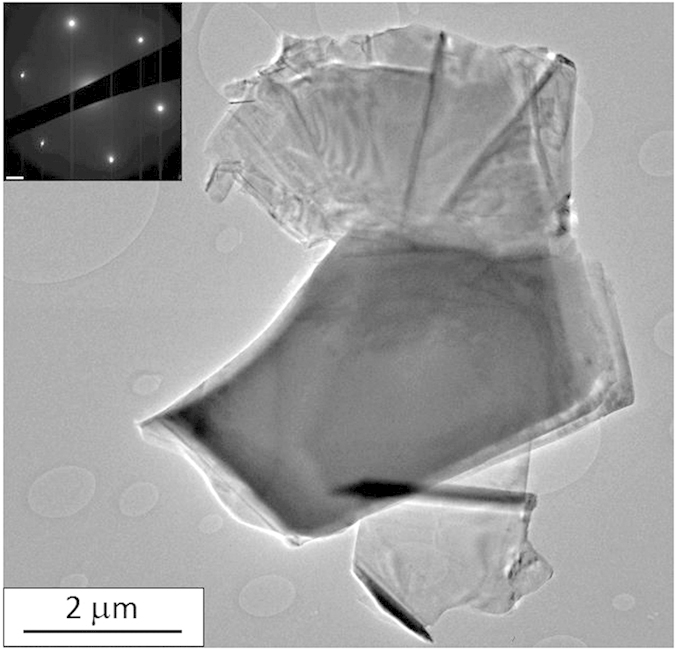
TEM BF-image showing a large crystal and its corresponding diffraction pattern (inset).

For a finer scale investigation, the h-BN raw pellet was scrubbed and mechanically exfoliated by the tape method, leading to 10 to 200 nm thick pieces with side size up to 30 μm (Fig. 3b). These BNNSs were deposited on a Si substrate, covered by a 80 nm SiO_2_ oxide layer in order to be characterized by Raman spectroscopy. Two spectra were recorded on two different BNNSs of the same sample differentiated by their contrast under optical microscope (Fig. 3a,b). Both spectra, shown in Fig. 3c, display the single peak of h-BN located at 1366 cm^−1^ with different intensities in relation to different BNNSs thicknesses. By AFM (Fig. 3c, inset), their thicknesses were measured at 13 nm and 114 nm. This result shows that mechanical exfoliation of h-BN leads to micrometric BNNSs of thicknesses down to few nanometers. Furthermore, the high crystalline quality of BNNS flakes is also confirmed by TEM (Fig. 4). Indeed, diffraction pattern associated with 5–30 μm BNNSs exhibited clearly hexagonal bright dots, which are representative of single crystals.

### Chemical exfoliation

**Figure 5:**
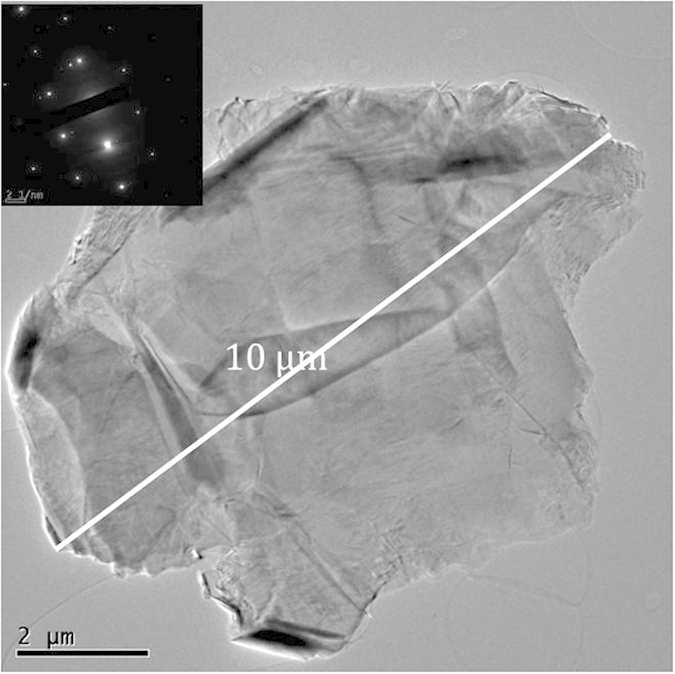
TEM BF-image showing a 10 μm BNNS and its corresponding diffraction pattern (inset).

**Figure 6:**
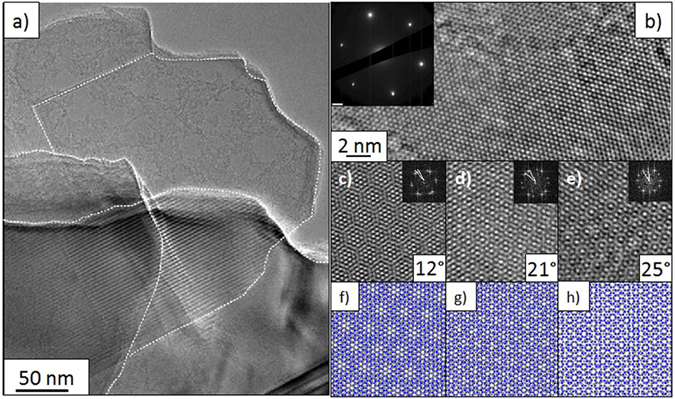
(**a**) BF-TEM micrograph of large h-BN sheets (10 μm) with Moiré patterns. Three misorientated single crystals were delimitated with dashed line; (**b**) typical hexagonal atomic arrangement of h-BN few layers, inset: corresponding SAED pattern; (**c–e**) particular atomic arrangement exhibiting hexagonal shaped Moiré patterns with angles misorientation of 12°, 21° and 25°, insets: corresponding FFT images; (**f–h**) modeling of two h-BN layers rotated of corresponding angles.

Finally, in order to perform a deeper structural investigation, single crystal flakes can be easily isolated into thinner BNNS by an appropriate ultrasonic exfoliation treatment in ethanol. We have studied the crystal and BNNSs size distribution, respectively before and after the chemical exfoliation, from SEM and optical images (see Supplementary Fig. S1 online). It is worth mentioning that such a size could still be increased by optimizing the exfoliation process. Figure 5 presents a TEM image recorded on an isolated BNNS measuring 10 to 15 μm. After exfoliation, their thickness slows down to several nanometers, which is approaching the targeted structure of graphene-like two-dimensional sheets. Then, the flatness of the BNNSs has been investigated by AFM (see Supplementary Fig. S2 online). In a typical study, the RMS (Root Mean Square) roughness has been determined to 0.7 nm. This value is similar to the one measured for standard silica. Even if we did not observe a lower value as it was shown in the work of Dean *et al.*^55^ we reasonably think that the flatness of our samples would be compatible with graphene deposition as substrate or to produce van der Waals heterostructures. Figure 6a shows homogeneous crystalline areas with Moiré patterns on extended zones confirming the high level of crystallinity. The selective area electron diffraction (SAED) pattern (Fig. 6b, inset), obtained from the high resolution image (Fig. 6b), shows six bright spots hexagonally distributed demonstrating the achievement of h-BN single crystals. This corresponding SAED pattern can be interpreted as the AA’ atomic stacking in the *c-*direction. Atomic layers can also be stacked in a more complex turbostratic structure, as demonstrated by the hexagonal-shaped Moiré patterns obtained on different samples area (Fig. 6c–e). Misorientations between h-BN layers similar to graphene can explain those Moiré patterns, which reveal themselves by multiple spots in corresponding Fast Fourier Transform images (Fig. 6c–e inset)^56^. Those images show two sets of hexagonal spots rotated with angles of 12°, 21° and 25° respectively. By modelling these misorientations with two BN monolayers, it is possible to match quite well corresponding FFT images (Fig. 6f–h). This relative rotation could be due to the stain induced by rolled structure, as already imaged for rotational stacking fault in few-layer graphene sheets^57^.

**Figure 7:**
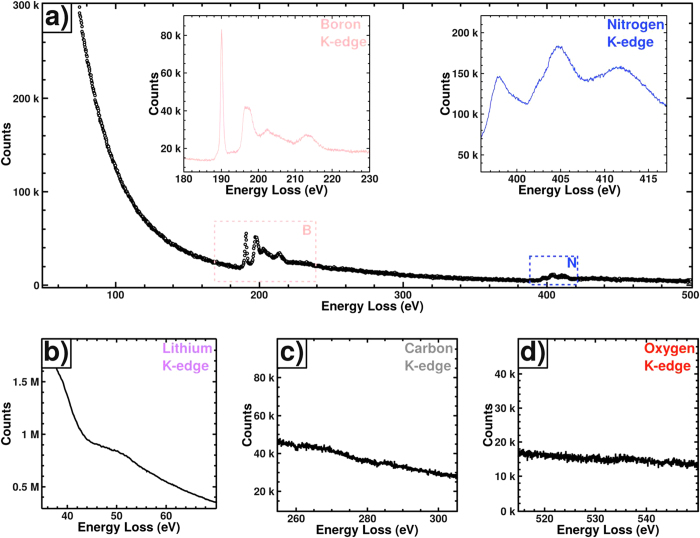
(**a**) Wide range EELS spectrum obtained on a BNNS, inset: high resolution spectra of B and N K-edge; (**b**) high resolution spectrum acquired around the K-edge of Li; (**c**) high resolution spectrum acquired around the K-edge of C and (**d**) high resolution spectrum acquired around the K-edge of O.

In order to get further information about the chemical composition, analysis by electron energy loss spectroscopy (EELS) was directly performed on thin BNNSs (Fig. 7). Only boron and nitrogen K-edges are recorded while no traces of lithium, carbon or oxygen is detected. This supplementary chemical analysis corroborates previous XPS results concluding on the excellent purity of samples: bulk, as well as BNNSs.

To summarize, the novelty of the method comes from the association of two synthesis processes: the PDCs route which is known to lead to high purity ceramic when a pure preceramic polymer is used and the SPS one that allows reaching high crystallinity to the final ceramic. The beneficial effect of SPS is obvious when comparing characteristics of the present BNNSs with the best ones synthesized by a single PDCs route^42^. For instance the crystalline quality is first significantly improved as shown by the FWHM Raman peaks measurement that decreased from 40 cm^−1^ for PDCs BNNSs to 7.7 cm^−1^ for the PDCs-SPS’BNNSs. Moreover, we demonstrated that applying an external current (by SPS) led to preferentially orientated flakes with much larger domains (up to 30 μm) than the ones obtained previously (1–2 μm). On the other hand, even if the NIMS laboratory may bring to the scientific community larger crystals (500 μm), their experimental procedure is more energy and time consuming. Indeed, it can be underlined that to reach GPa pressure conditions, a specific and dedicated instrument (diamond anvil) is required, which limits the entire size of the sample and is not easy to access. Summarized comparative data are gathered as Supplementary Table S2 online.

Finally, the quality of our samples may open new perspectives for many applications as graphene substrate or to produce van der Waals heterostructures.

## Conclusions

To conclude, we have demonstrated that a large amount of self-standing h-BN few-layers can be obtained by exfoliation of highly crystallized h-BN bulk material, the latter being prepared using a relevant dual synthesis method involving the PDCs route and the SPS process. This original method offers a new alternative way to generate high quality h-BN crystals as new source of supply for BBNSs.

## Methods

A dispersion of lithium nitride (Li_3_N, Aldrich) micro powders (5 wt%) in liquid polyborazilene (PBN), was prepared following a procedure described elsewhere^42^. This dispersion was slowly heated in a Schlenk tube under argon from room temperature to 200 °C and held at 200 °C for 1 h leading to a solid-state polymer avoiding further oligomer evaporation. The obtained white powder (82% mass yield) was then placed into an alumina crucible to be heated under N_2_ at 650 °C (1 °C.min^−1^), and kept at this final temperature for 1 h. In a gloves box, the SPS graphite die covered with a papyex® was filled with the preceramic powder and then transferred into the SPS chamber (HP D 25, FCT System) in-between the two punches and compressed at room temperature under uniaxial load until 90 MPa. SPS heating (10 ms pulses ON and 5 ms OFF) was then conducted up to 1800 °C keeping the normal load constant. During the 1 h dwell temperature the current was 770A and the vacuum in the SPS chamber was 0.15 mbar. The annealing process was held at this maximum temperature to complete the BN ceramization reaction and the densification process. Removing the protective papyex, a white pellet (300 mg) was finally obtained. SEM observations were carried out at very low accelerating voltage (200 V) using a SUPRA Zeiss microscope. Raman spectra were acquired in a Horiba Jobin-Yvon ARAMIS spectrometer equipped with a 1800 lines/nm grating, using an excitation wavelength of 532 nm and a x50 objective (the main Raman band of the silica peak is located at 520.1 cm^−1^). XPS measurements were done using a PHI Quantera SXM instrument equipped with a 180 hemispherical electron energy analyzer and a monochromatized Al Kα (1486.6 eV) source operated at 15 kV and 4 mA. Analyses were performed on the raw sample and after a 1 μm abrasion using a 4 keV Ar+ sputtering. XRD patterns were recorded with a Phillips PW 1830/40 diffractometer, with a CuK radiation source. BNNSs are then prepared by chemical (in ethanol, ultrasonication: 1 min at 25 W, Hielscher UP400S and centrifugation) or physical (tape) exfoliation. A droplet of the solution was simply deposited onto a 300 mesh holey carbon copper grid in order to perform TEM observations (JEOL 2100F and 2010F microscopes operating at 200 kV). EELS spectra were recorded using a JEOL JEM-ARM200F Cold FEG, operating at 200 kV.

## Additional Information

**How to cite this article**: Yuan, S. *et al.* Pure & crystallized 2D Boron Nitride sheets synthesized via a novel process coupling both PDCs and SPS methods. *Sci. Rep.* **6**, 20388; doi: 10.1038/srep20388 (2016).

## Supplementary Material

Supplementary Information

## Figures and Tables

**Table 1:** XRD data (a) as reported for h-BN in the JCPDS file number 01-073-2095; (b) obtained from the pattern recorded on the raw sample for an X-Ray analysis of the pellet surface and (c) obtained from the pattern recorded on the raw sample for an X-Ray analysis of the pellet cross section.

	**(a) Data from the JCPDS 01-073-2095**			**(b) Data from XRD pattern recorded (⊥)**			**(c) Data from XRD pattern recorded (∥)**		
(h,k,l)	2θ (°)	d (Å)	R. I.* (%)	2θ (°)	d (Å)	R.I. (%)	2θ (°)	d (Å)	R.I. (%)
(002)	26.75	3.33	100	26.77	3.33	100	26.56	3.353	100
(100)	41.61	2.17	14	41.60	2.17	60	41.57	2.17	2.2
(101)	43.87	2.06	4.5	43.88	2.06	17	43.12	2.1	1
(102)	50.16	1.82	12	50.15	1.82	23	50.09	1.82	1
(004)	55.11	1.67	5	55.10	1.67	6	54.64	1.68	2.7
(103)	59.54	1.55	1	59.52	1.55	2	59.43	1.55	0.1
(104)	71.35	1.32	2.5	71.32	1.32	3	71.31	1.32	0.1
(110)	75.94	1.25	4	75.93	1.25	20	75.87	1.25	1
(112)	82.19	1.17	6	82.15	1.17	16	82.08	1.17	0.1

^*^Relative intensity normalized by the (002) diffraction peak.
