# Supplementary material for: Pure & crystallized 2D Boron Nitride sheets synthesized via a novel process coupling both PDCs and SPS methods
Source: Sci Rep. 2016 Feb 4;6:20388. doi: 10.1038/srep20388 (PMC4740893; doi:10.1038/srep20388)
Supplement: Supplementary Information [file srep20388-s1.doc]

Supplementary Information

Pure & crystallized 2D Boron Nitride sheets synthesized via a novel process coupling both PDCs and SPS methods

Sheng Yuan, Sébastien Linas, Catherine Journet, Philippe Steyer, Vincent Garnier, Guillaume Bonnefont, Arnaud Brioude and Bérangère Toury


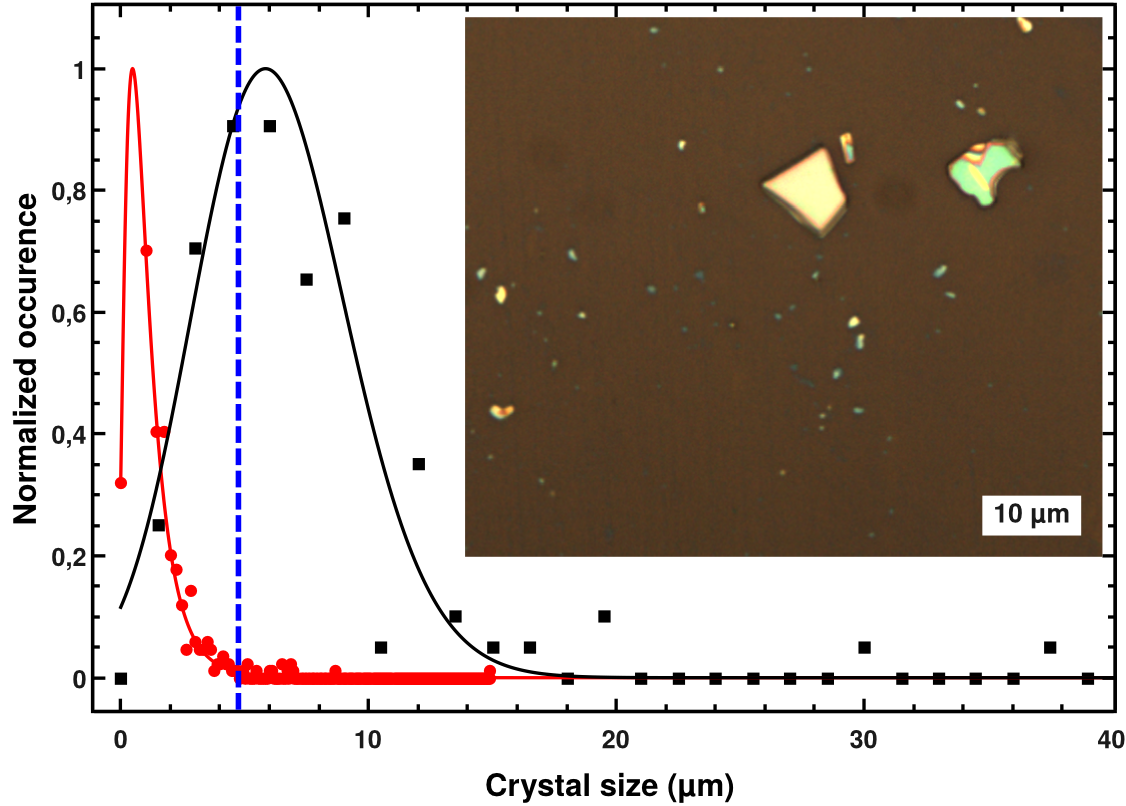
**Supplementary Figure S1.** Graph showing the crystals and BNNSs size distribution, respectively before (black) and after (red) chemical exfoliation, from SEM and optical images. Dashed blue line corresponds to the crystals area range (up to 25 µm^2^) which is suitable for electronic lithography processes involved in electronic transport applications based on graphene.


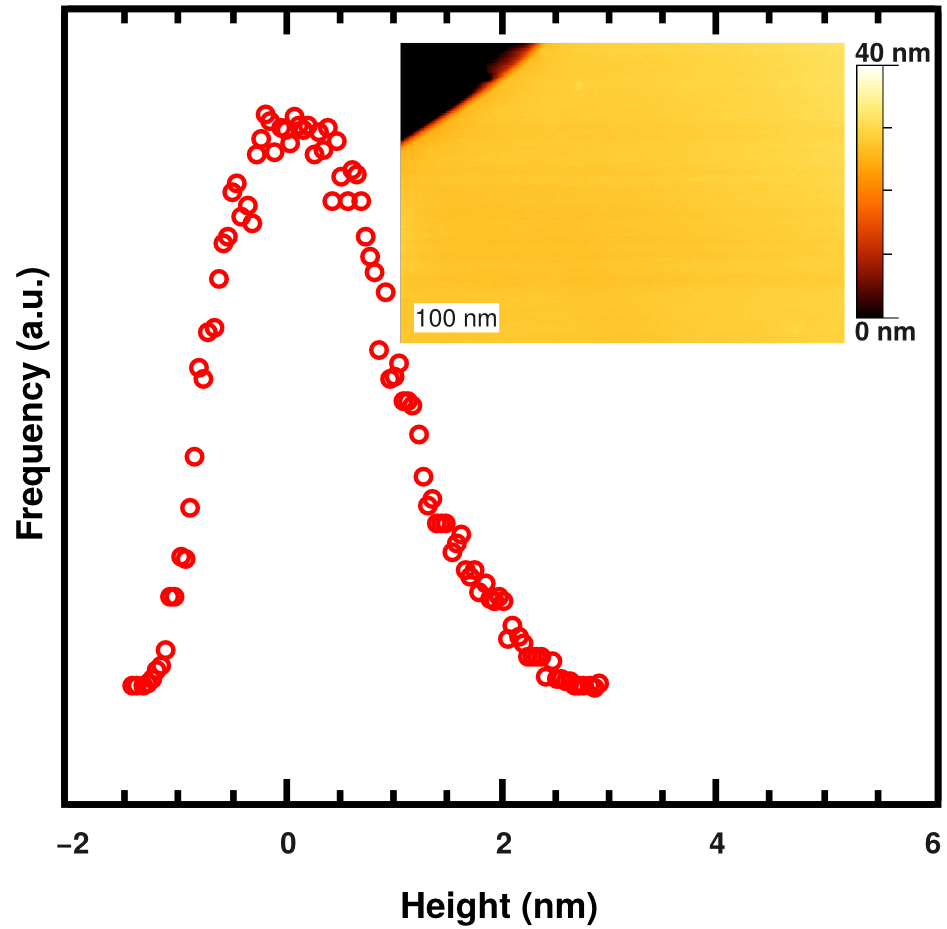


**Supplementary Figure S2.** Histogram of the height distribution (surface roughness) measured by AFM for h-BN deposited on SiO_2_ (inset).

|  | worldwide benchmark - NIMS^1,2^ | Previous work^3^ | Present work |
| --- | --- | --- | --- |
| Max synthesis temperature | 2100°C | 1400°C | 1800°C |
| Max synthesis pressure | 5.5GPa | No pressure | 90MPa |
| Total heating time | 20-80h | 1h | 1h |
| Crystals size (maximum) | 500µm | 1-2µm | 30µm |
| Raman FWHM | 8 cm^-1^ | 40 cm^-1^ | 7.7 cm^-1^ |

**Supplementary Table S2:** Comparative data on experimental synthesis conditions and BNNSs characterizations obtained with different BNNSs source

REFERENCES

1. Watanabe, K., Taniguchi, T. & Kanda, H. Direct-bandgap properties and evidence for ultraviolet lasing of hexagonal boron nitride single crystal. *Nat. Mater.* **3,** 404–409 (2004).

2. Kubota, Y., Watanabe, K., Tsuda, O. & Taniguchi, T. Deep Ultraviolet Light-Emitting Hexagonal Boron Nitride Synthesized at Atmospheric Pressure. *Science* **317,** 932–934 (2007).

3. Yuan, S., Toury, B., Journet, C. & Brioude, A. Synthesis of hexagonal boron nitride graphene-like few layers. *Nanoscale* **6,** 7838–7841 (2014).
